# Cardiometabolic outcome of MyBFF@school intervention program among primary schoolchildren: a cluster randomized controlled trial

**DOI:** 10.1186/s12889-025-23546-x

**Published:** 2025-07-24

**Authors:** Muhammad Yazid Jalaludin, Ruziana Mona Wan Mohd Zin, Farah Aqilah Roslan, Fazliana Mansor, Fuziah Md. Zain, Janet Yeow Hua Hong, Nur Zati Iwani Ahmad Kamil, Abqariyah Yahya, Zahari Ishak, Rusidah Selamat, Abdul Halim Mokhtar

**Affiliations:** 1 Department of Pediatrics, Faculty of Medicine, Universiti Malaya, Kuala Lumpur, Wilayah Persekutuan Kuala Lumpur 50603 Malaysia; 2 Endocrine and Metabolic Unit, Institute for Medical Research, National Institute of Health (NIH), Ministry of Health Malaysia, Setia Alam, Shah Alam, Selangor 40170 Malaysia; 3 Department of Pediatrics, Putrajaya Hospital, Ministry of Health Malaysia, Jalan P9, Pusat Pentadbiran Kerajaan Persekutuan Presint 7, Putrajaya, Wilayah Persekutuan 62250 Malaysia; 4 Department of Social and Preventive Medicine, Faculty of Medicine, Universiti Malaya, Kuala Lumpur, Wilayah Persekutuan Kuala Lumpur 50603 Malaysia; 5 Faculty of Social Sciences & Liberal Arts, UCSI University, No.1, Jalan UCSI, UCSI Heights, Cheras, Kuala Lumpur, 56000 Malaysia; 6 Nutrition Division, Ministry of Health Malaysia, Level 1, Block E3, Complex E, Federal Government Administrative Centre, Putrajaya, Wilayah Persekutuan 62590 Malaysia; 7 Department of Sports Medicine, Faculty of Medicine, Universiti Malaya, Kuala Lumpur, Wilayah Persekutuan Kuala Lumpur 50603 Malaysia; 8 Faculty of Sports and Exercise Science, Universiti Malaya, Kuala Lumpur, Wilayah Persekutuan Kuala Lumpur 50603 Malaysia

**Keywords:** Insulin resistance, Diabetes, Blood pressure, Lipid profile, School-based intervention, Childhood obesity

## Abstract

**Background:**

The increasing prevalence of obesity is associated with the increase in type 2 diabetes, dyslipidemia and hypertension among children. The MyBFF@school study aimed to assess the effectiveness of a 6-month intervention program on the cardiometabolic markers of the overweight and obese primary schoolchildren.

**Methods:**

MyBFF@school is a cluster randomized controlled trial involving 23 of 1196 government schools in Malaysia. Schoolchildren aged $9-11$ years with a body mass index (BMI) adjusted for gender and age (BMI *z*-score) > + 1 SD were recruited. This program (incorporating physical activity, nutrition and psychological components) was conducted during school hours. The effect of the intervention on cardiometabolic status was determined both within and between groups using repeated analysis of variance together with analysis of covariance. Out of 1397 schoolchildren, 683 (intervention = 390 and control = 293) had fasting blood taken at baseline and month-6.

**Results:**

At the end of the intervention, both the intervention and control groups had significantly increased BMI, however, when adjusted for gender and age, the BMI *z*-score in the intervention group showed no changes, whereas the control group had a significant increase in BMI *z*-score (mean difference of 0.03, 95% CI: 0.04, 0.06, *p* = 0.026). Fasting insulin showed significant reduction in the intervention group (˗3.16 μU/mL (95% CI: ˗4.07, ˗2.25), *p* < 0.001) vs control (˗1.05 μU/mL (95% CI: ˗2.28, 0.18), *p* = 0.095) (*p* < 0.001 between groups). A significantly greater reduction of HOMA-IR was observed in the intervention group [˗0.41 (95% CI: ˗ 0.63, ˗0.19), *p* < 0.001] vs control [˗0.17 (95% CI: ˗0.45, 0.11), *p* = 0.231)] (*p* < 0.001 between groups). HDL-C also showed significant increment of 0.34 mmol/L [(95% CI: 0.32, 0.36), *p* < 0.001] in the intervention and 0.11 mmol/L [(95% CI: 0.08, 0.14), *p* < 0.001] in the control group (*p* < 0.001 between groups). A significant reduction of TG: HDL-C ratio was observed in both the intervention [˗0.17 mmol/L (95% CI: ˗0.21, ˗0.12), *p* < 0.001] and control groups [˗0.12 mmol/L (95% CI: ˗0.17, ˗0.07), *p* < 0.001] (*p* = 0.524 between groups).

**Conclusion:**

MyBFF@school resulted in significant improvements of fasting insulin, HOMA-IR and HDL-C in the intervention group. We recommend MyBFF@school program to improve the cardiometabolic markers of the overweight and obese primary schoolchildren.

**Trial registration:**

Clinical trial number: NCT04155255, November 7, 2019 (Retrospective registered). National Medical Research Register: NMRR-13-439-16563. Registered July 23, 2013. The Medical Research and Ethics Committee (MREC), Ministry of Health Malaysia and Educational Planning and Research Division (EPRD), Ministry of Education Malaysia approved the intervention program. It was funded by the Ministry of Health Malaysia.

## Background

The prevalence of obesity in Malaysia has increased substantially in recent years. The 2011 National Health and Morbidity Survey (NHMS) Malaysia reported that 3.9% (0.3 million) of those below 18 years of age were obese, and this figure increased to 14.8% by 2019 [1]. The etiology of childhood obesity is multifactorial, including contributions from the host (genetic and learned behavior) and agent (energy imbalance), together with aspects of the modern sedentary lifestyle (e.g., passive overeating) and various socio-cultural economic influences [2, 3]. If left untreated, obesity in young people can lead to chronic diseases, such as type 2 diabetes (T2D), dyslipidemia, and cardiovascular disease, and eventually to premature mortality [4]. Despite the rapidly increasing prevalence of childhood obesity reported worldwide, an intervention to combat obesity and its related comorbidities remains challenging [5].

T2D was traditionally viewed as an adult-onset disease. However, over the last two decades, scientific literature has shown a global and dramatic increase of the incidence of T2D in youth [6], secondary to the coincident pandemic of childhood obesity [7]. Insulin resistance is a common feature of childhood obesity and is also a major risk factor for T2D development in obese children [8, 9]. Insulin resistance (IR) can be identified clinically (presence of acanthosis nigricans) or by the increased in the homeostatic model assessment of insulin resistance (HOMA-IR) index. Insulin resistance is essentially characterised by the decrease in the capacity of attaining normal plasma insulin concentrations, promoting adequate peripheral glucose uptake, maintaining liver glycogenesis in balance, and inhibiting the production of very-low-density lipoprotein (VLDL) [10]. Progression of IR can lead to metabolic syndrome (MetS), non-alcoholic fatty liver disease (NAFLD), and T2DM [11]. Among overweight and obese Malaysian children, 53.8% had acanthosis nigricans while 49.4% of pubertal children and 66.2% pre-pubertal children had high HOMA-IR [12]. Recently, triglycerides to high-density lipoprotein cholesterol (TG: HDL-C) ratio has been suggested as an alternative simple marker of insulin resistance [12–15]. Dyslipidemia is a major risk in individuals for whom atherosclerosis affects medium–large arteries and may cause ischemia in the brain, heart, or legs [16]. Furthermore, both randomized trials [17] and large observational studies [18, 19] have shown that elevated total cholesterol, triglycerides, low-density lipoprotein cholesterol (LDL-C), and low levels of high-density lipoprotein cholesterol (HDL-C) in the blood are associated with an increased risk of cardiovascular disease. Moreover, Himah et al. [19] showed that a higher risk of dyslipidemia is associated with obese children (RR = 5.2, 95% CI 4.2, 5.9) than non-obese children.

Pediatric hypertension is among the strongest predictors of adult hypertension, which increases cardiovascular mortality risk [20]. This problem is believed to be increased as a result of the epidemic of childhood overweight and obesity. However, hypertension and pre-hypertension are under-diagnosed in the pediatric population. A recent study by Sreeramareddy et al. [21] reported that the prevalence of pre-hypertension in overweight/obese children in Selangor, Malaysia was 12.2% and hypertension was 13.4%, and that the mean systolic and diastolic blood pressure was higher among overweight/obese children as compared to normal children.

School has a long-lasting influence on most children as a platform for health education regarding diet, physical activity, and other healthy behaviors. Veugelers and Fitzgerald [22] demonstrated the efficacy of school-based programs in mitigating childhood obesity and showed that schools play a crucial role in future health by educating on healthy diets and physical activity. Moreover, a review by Sharma [23] stated that intervention programs carried out in upper elementary and lower middle schools were successful in preventing and treating childhood obesity. The Cardiovascular Health in Children Study I (CHIC I) showed a significant increase in self-reported physical activity in intervention groups. After eight weeks of intervention, intervention groups exhibited a reduction in total cholesterol, an increase in aerobic power, and a reduction of body fat [24].

Nevertheless, the amount of data on effective intervention programs targeting childhood obesity is still limited. Previous studies mainly focused on weight loss intervention involving adult clinical settings. To the best of our knowledge, the My Body is Fit and Fabulous at School (MyBFF@school) initiative is the first large-scale school-based intervention study involving overweight and obese schoolchildren aged 9–11 years in Malaysia. The objective of this study is to evaluate the effect of school-based intervention on cardiometabolic markers such as blood glucose, insulin resistance, lipid profiles, and blood pressure status after six months of intervention.

## Methods

### Study design and participants

**Fig. 1:**
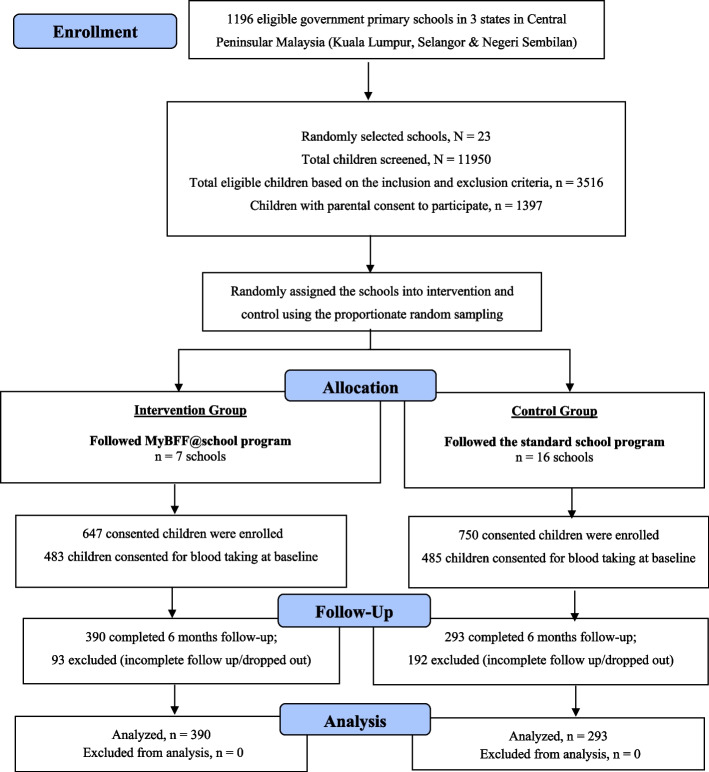
CONSORT diagram for clinical component in MyBFF@school

This study is part of MyBFF@school initiative. MyBFF@school is a multi-component intervention study (physical activity, nutrition, and psychology) involving overweight and obese schoolchildren. This is a school-based, cluster randomized controlled trial that included public primary schools in the central region of Peninsular Malaysia, specifically in Selangor, Kuala Lumpur, and Negeri Sembilan. Schools eligible for participation were randomly selected using proportionate random sampling to ensure a representative sample of the multi-ethnic population. The schools were stratified by type (national or vernacular primary schools) and location (urban or rural). The selected schools were then randomly assigned to either the intervention group or the control group. In total, 23 primary schools were assigned, with 7 schools in the intervention group and 16 schools in the control group. The details of school selection and group allocation are provided in Fig. 1. The intervention study was conducted between February 2016 and August 2016. MyBFF@school program incorporated physical activity in the form of small-sided games (SSG), nutrition, and psychology components. SSG (football, handball, and fun games) sessions were conducted for 30 min as allocated in the school curriculum. Two sessions were organized per week; therefore, a total of 60 min per week was spent on SSG activities. Nutrition education intervention (NEI) utilized the nutrition education modules (NEM) adapted from the Malaysian Childhood Obesity Treatment Trial (MASCOT)’s modules, and was conducted on alternate week with psychology component for 24 weeks by trained research assistants (45–60 min per session). The main themes in the psychology modules were self-esteem, friendship, assertiveness, and positive thinking for a healthy lifestyle and stress management. Motivational talks and interactive activities were the two main mechanisms used to achieve the objectives, followed by a reflection session at the end of the activities. All components were carried out during physical education periods and co-curriculum activities in the school during school hours. The research team monitored the schoolchildren on a monthly basis, but formal assessment was performed at baseline, three and six months.

On the other hand, the schoolchildren in the control group followed the standard school program. In Malaysia, a standard health curriculum for primary schoolchildren is delivered from the age of seven (Year 1) until the age of 12 (Year 6). It is incorporated into the school curriculum as part of health education and is mandatory in all government schools. The standard contents are nutrition, mental health, personal hygiene, reproductive health, and selected diseases. This health education subject is being taught over 35 sessions (30 min each) per year. However, the nutrition component consists of only five sessions (15%), equivalent to 150 min per year. In addition, no hands-on or interactive session is included in the nutrition component. Apart from that, in the existing standard school program, physical activity sessions are allocated twice a week with 30 min for each session while co-curricular activity is allocated only one hour per week.

The inclusion criteria for selecting the schoolchildren were children aged 9–11 years old, overweight or obese, with a BMI z-score of more than + 1 SD based on the WHO 2007 Growth Reference. The exclusion criteria were schoolchildren with BMI z-score below and/or equal to + 1 SD, with physical or mental disability, medical conditions that prevented their participation in moderate to vigorous physical activities, co-morbidities that may interfere with the study (such as T2D, hypertension, nephritic syndrome, epilepsy, congenital heart disease and skeletal anomalies), or a requirement for steroids, anti-epileptic treatments, or methylphenidate.

The study design and methodology are fully elaborated by Mokhtar et al. [25]. A total of 11,950 primary schoolchildren aged 9–11 years were screened, of which 3516 overweight and obese children were invited to join the study. 1397 children consented to their participation in the study, of whom 647 were placed in the intervention group and 750 were placed in the control group. At baseline, 968 children underwent blood sampling (intervention = 483 and control = 485). Analyses were made on samples from children in both the intervention and control groups, who had complete blood data at both baseline and after six months (Fig. 1).

### Demographic and anthropometric measurements

Age, gender, ethnicity, school locality, and pubertal status were documented by medical officers, and this information was recorded in each child’s booklet. Body mass index (BMI) was calculated by dividing weight in kilograms by the square of height in meters (kg/m^2^). BMI category was classified based on the World Health Organization (WHO) BMI z-score criteria, which is overweight, obese, and morbidly obese [26]. The z-score of BMI distribution was calculated using WHO AnthroPlus 2007 software, with the designations of overweight, > + 1.0 SD; obese, > + 2.0 SD; and morbidly obese, > + 3.0 SD. Pubertal/sexual maturity was self-assessed following Tanner’s staging scale criteria [27, 28].

### Clinical measurements

Blood pressure readings were obtained manually by trained staff, using a mercury sphygmomanometer (Accoson, UK) with an appropriate cuff size for each individual. Children were seated with the right upper arm positioned at the heart level and their feet flat on the ground. The reading was taken twice, with a 5 min interval between measurements to improve accuracy, and the mean value was recorded.

Trained nurses or medical officers took venous blood samples after a minimum of 8 h of fasting. Two milliliters (mL) of blood was collected in sodium fluoride tubes for glucose measurements, and another 5 mL was collected in plain test tubes to measure insulin levels and lipid profiles (total cholesterol, triglycerides, HDL-C, and LDL-C). All blood samples were labeled and transported in an icebox to the central laboratory at the Institute for Medical Research within 2 h of collection. Plasma glucose and serum total cholesterol, triglycerides, HDL-C, and LDL-C were analyzed by Randox Laboratories (Antrim, UK) using an auto chemical analyzer (Dirui CS-400, China). Fasting insulin levels were measured using an automated enzyme immunoassay analyzer (TOSOH AIA-360, Japan).

### Definition of measures

Diabetes mellitus is determined according to fasting plasma glucose levels using indications of normal (< 5.6 mmol/L), pre-diabetes (5.6–7.0 mmol/L), and diabetes (≥ 7.0 mmol/L) [26]. HOMA-IR is calculated by multiplying the value of fasting insulin and fasting plasma glucose before dividing this value by 22.5 [29]. The categories of HOMA-IR were based on pubertal status as pubertal transition is associated with insulin resistance [30]. For pre-pubertal children, the categories are insulin sensitive (< 2.6) and insulin resistance (≥ 2.6) [31]. For pubertal children, the categories are insulin sensitive (< 4.0) and insulin resistance (≥ 4.0) [32]. TG: HDL-C ratios are calculated by dividing triglyceride levels by HDL-C levels. For lipid profiles, the cut-off values for high total cholesterol, high triglycerides, low HDL-C, and high LDL-C were 5.20, 1.70, 1.03, and 2.84 mmol/L, respectively. Children were categorized based on updated definitions of blood pressure categories by the American Heart Association [33]. These categories are as follows: normal (< 90th percentile), elevated (≥ 90th percentile to < 95th percentile), stage 1 (≥ 95th percentile to < 95th percentile + 12 mmHg), and stage 2 (≥ 95th percentile + 12 mmHg).

### Statistical analysis

All data analyses were performed using IBM Statistical Package for Social Sciences version 24. Baseline characteristics were determined for both the intervention and control groups. Continuous data were presented as the mean and standard deviation, whereas categorical data were described in frequency and percentage. Independent t-tests for continuous variables and chi-square tests for categorical variables were used to distinguish differences between the characteristics of study groups. Differences within groups were determined using repeated measures of analysis of variance (ANOVA), whereas differences of mean changes between groups were measured using repeated measures of analysis of covariance (ANCOVA) with adjusted gender and baseline variables. *P*-values of < 0.05 were considered statistically significant.

## Results

Figure 1 shows the CONSORT diagram of MyBFF@school program. Twenty-three primary schools participated in this study and were randomly assigned to either intervention (seven schools) or control (16 schools) arm. A total of 1397 primary schoolchildren aged 9–11 years old from these schools were enrolled in this study. Blood taking was done at baseline and month-6 post-intervention. Since blood taking was optional, 968 children consented and completed blood collection at baseline (483 children from intervention school and 485 children from control school) and 683 children completed blood taking at month-6 (390 children from intervention school and 293 children from control school). A total of 93 and 192 children from intervention and control groups respectively, were excluded from the analysis due to loss to follow-up.

Table 1 shows socio-demographic and anthropometric measures of the intervention and control groups at baseline. At this point, there was no significant difference between the two groups in age, gender, or pubertal status. The mean (SD) ages of the intervention and control groups were 9.85 (0.82) and 9.91 (0.82) years, respectively. The majority of the children in both groups were Malay, followed by Chinese, Indian, and other ethnic groups, which is consistent with the ethnicity distribution in Malaysia. There were slightly more children from the rural schools (50.5%) in the intervention group than in the control schools (39.9%). The control group had a slightly higher BMI z-score (2.40 ± 0.82) than the intervention group (2.24 ± 0.78). Both groups had a high percentage of morbidly obese individuals, with 15.1% and 21.8%, respectively.

**Table 1:** Socio-demographic and anthropometric measures of MyBFF@school participants in primary school

Characteristics	Intervention (*n* = 390)	Control (*n* = 293)	*P*-value
Demographic			
Age, mean ± SD	9.85 ± 0.82	9.91 ± 0.82	0.311
Gender, n (%)			
Boys	192 (49.2)	151 (51.5)	0.589
Girls	198 (50.8)	142 (48.5)	
Ethnicity, n (%)			
Malay	294 (75.4)	204 (69.6)	0.024
Chinese	51 (13.1)	59 (20.1)	
Indian	27 (5.9)	24 (8.2)	
Others	18 (4.6)	6 (2.0)	
School location, n (%)			
Urban	193 (49.5)	176 (60.1)	0.007
Rural	197 (50.5)	117 (39.9)	
Pubertal status, n (%)			
Pre-pubertal	249 (63.8)	195 (67.2)	0.371
Pubertal	141 (36.2)	95 (32.8)	
Anthropometric Measures			
BMI (kg/m^2^), mean ± SD	23.48 ± 3.65	24.24 ± 3.82	0.009
BMI z-score, mean ± SD	2.28 ± 0.79	2.40 ± 0.82	0.011
BMI categories, n (%)			
Overweight	159 (40.8)	98 (33.4)	0.037
Obese	172 (44.1)	131 (44.7)	
Morbidly obese	59 (15.1)	64 (21.8)	

*P*-value was calculated using an independent *t*-test for continuous variables and chi-square test for categorical variables

Pubertal status missing data: control (*n* = 3)

*Abbreviations*: *SD* standard deviation, *BMI* body mass index

The distribution of diabetes, insulin resistance, lipid profiles, and blood pressure statuses at baseline is shown in Table 2. Biochemical profiles of the intervention and control groups were comparable at baseline, except for those of fasting insulin, HOMA-IR, HDL-C, LDL-C, and blood pressure. At baseline, the prevalence rates of children with pre-diabetes in the intervention and control groups were 0.5% and 2.4%, respectively. In the intervention group, pre-pubertal children with insulin resistance accounted for 39.4% of the tested individuals, and pubertal children with insulin resistance accounted for 24.8%. Pre-pubertal and pubertal children with insulin resistance in the control group accounted for 50.5% and 35.8% of the sample, respectively. Lipid profile status showed that the intervention group had a higher prevalence of high triglycerides and low HDL-C, whereas a higher prevalence of high total cholesterol and high LDL-C was seen in the control group. For blood pressure status, 5.6% of children in the intervention group had elevated blood pressure, 10.9% had stage 1 hypertension, and 0.3% showed stage 2 hypertension. Meanwhile, children with elevated blood pressure, stage 1 hypertension, and stage 2 hypertension in the control group accounted for 12.5%, 13.5%, and 1.1%, respectively.

**Table 2:** Baseline distribution of diabetes, insulin resistance, lipid profiles, and blood pressure statuses among MyBFF@school participants in primary school

Characteristics	Intervention (*n* = 390)	Control (*n* = 293)	*P*-value
Diabetes			
*Fasting plasma glucose* (mmol/L), mean ± SD	4.77 ± 0.33	4.72 ± 0.38	0.069
Normal (< 5.6 mmol/L), n (%)	388 (99.5)	286 (97.6)	0.043
Pre-diabetes (5.6–7.0 mmol/L), n (%)	2 (0.5)	7 (2.4)	
Diabetes (≥ 7.0 mmol/L), n (%)	0	0	
Insulin resistance			
^a^*Fasting insulin* (μU/mL), mean ± SD	13.23 ± 9.98	15.55 ± 9.98	0.003
^a^*HOMA-IR*, mean ± SD	2.84 ± 2.29	3.28 ± 2.13	0.011
Pre-pubertal insulin sensitive (< 2.6), n (%)	151 (60.6)	98 (49.5)	0.021
Pre-pubertal insulin resistance (≥ 2.6), n (%)	98 (39.4)	100 (50.5)	
Pubertal insulin sensitive (< 4.0), n (%)	106 (75.2)	61 (64.2)	0.080
Pubertal insulin resistance (≥ 4.0), n (%)	35 (24.8)	34 (35.8)	
Lipid profile			
*Total cholesterol* (mmol/L), mean ± SD	4.16 ± 0.71	4.24 ± 0.66	0.134
Normal (< 5.2 mmol/L), n (%)	360 (92.3)	266 (90.8)	0.488
High (≥ 5.2 mmol/L), n (%)	30 (7.7)	27 (9.2)	
*Triglycerides* (mmol/L), mean ± SD	0.99 ± 0.48	0.99 ± 0.45	0.927
Normal (< 1.7 mmol/L), n (%)	360 (92.3)	273 (93.2)	0.767
High (≥ 1.7 mmol/L), n (%)	30 (7.7)	20 (6.8)	
*HDL-C* (mmol/L), mean ± SD	1.10 ± 0.23	1.17 ± 0.24	< 0.001
Normal (≥ 1.03 mmol/L), n (%)	229 (58.7)	206 (70.3)	0.002
Low (< 1.03 mmol/L), n (%)	161 (41.3)	87 (29.7)	
*LDL-C* (mmol/L), mean ± SD	2.74 ± 0.80	3.10 ± 0.77	< 0.001
Normal (< 2.84 mmol/L), n (%)	229 (58.7)	112 (38.2)	< 0.001
High (≥ 2.84 mmol/L), n (%)	161 (41.3)	181 (61.8)	
*TG:HDL-C* (mmol/L), mean ± SD	0.95 ± 0.54	0.90 ± 0.48	0.189
Blood pressure			
*Systolic* (mmHg), mean ± SD	98.43 ± 10.80	102.77 ± 9.49	< 0.001
*Diastolic* (mmHg), mean ± SD	60.88 ± 9.74	66.09 ± 8.25	< 0.001
Normal (< 90th percentile), n (%)	312 (83.2)	205 (73.0)	0.004
Elevated blood pressure (≥ 90th percentile to < 95th percentile), n (%)	21 (5.6)	35 (12.5)	
Stage 1 HTN (≥ 95th percentile to < 95th percentile + 12 mmHg), n (%)	41 (10.9)	38 (13.5)	
Stage 2 HTN (≥ 95th percentile + 12 mmHg), n (%)	1 (0.3)	3 (1.1)	

*P*-value was calculated using independent *t*-test for continuous variables and chi-square test for categorical variables

Outliers (DM) were excluded from the analysis (*n* = 9)

*Abbreviations*: *SD* standard deviation, *HOMA-IR* homeostatic model assessment of insulin resistance, *HDL-C* high-density lipoprotein cholesterol, *LDL-C* low-density lipoprotein cholesterol, *TG: HDL-C* triglycerides to high-density lipoprotein cholesterol ratio, *HTN* hypertension

^a^Intervention (*n* = 381), control (*n* = 251)

Table 3 shows cardiometabolic marker changes within and between groups after six months of intervention. At the end of the intervention, both the intervention and control groups had significantly increased body mass index (BMI), with the intervention group showing a significantly smaller increase compared to the control group (mean difference of −0.53 kg/m2 (95% CI: −1.05, −0.01), *p* = 0.044). However, when adjusted for gender and age, the BMI z-score in the intervention group showed no changes, whereas the control group had a significant increase in BMI z-score at the end of the intervention (mean difference of 0.03, 95% CI: 0.04, 0.06, *p* = 0.026). Both groups showed an improvement in fasting insulin, with a significant reduction of 3.16 μU/mL (95% CI − 4.07, − 2.25) in the intervention group and a reduction of 1.05 μU/mL (95% CI − 2.28, 0.18) in the control group. A significantly greater reduction of HOMA-IR was observed in the intervention group [− 0.41 (95% CI − 0.63, − 0.19)] but not in the control group [− 0.17 (95% CI − 0.45, 0.11)]. However, the reduction was nonetheless significant when compared between the two groups. Fasting plasma glucose was significantly increased post-intervention. In the case of lipids, HDL-C showed a significant improvement with an increment of 0.34 mmol/L (95% CI 0.32, 0.36) in the intervention group and 0.11 mmol/L (95% CI 0.08, 0.14) in the control group, with a significant difference detected between the two groups. However, significant increases in total cholesterol and LDL-C in both groups were also seen. A significant reduction of TG: HDL-C ratio was observed in the intervention [− 0.17 mmol/L (95% CI − 0.21, − 0.12)] and control [− 0.17 mmol/L (95% CI − 0.17, − 0.07)] groups, but no significant difference between groups was detected. A reduction in triglycerides in the control group [− 0.04 mmol/L (95% CI − 0.09, 0.00)] was observed, albeit without statistical significance, but there was a significant increase of 0.07 mmol/L (95% CI 0.02, 0.11) in the intervention group. Systolic blood pressure increased in both the intervention and control groups, but between-group analysis showed no significant difference. Although diastolic blood pressure in the intervention group had not significantly changed after intervention, between-group analysis showed significant differences between the intervention and control groups.

**Table 3:** Changes of BMI and cardiometabolic markers within and between groups after six months of intervention

Measures	Group	Baseline (mean ± SD)	Month 6 (mean ± SD)	Within group, MD (95% CI)	*P*-value^a^	Between group, MD (95% CI)	*P*-value^b^
BMI (kg/m^2^)	Intervention (*n* = 390)	23.48 ± 3.65	24.24 ± 3.84	0.62 (0.51, 0.73)	< 0.001	−0.53 (−1.05, −0.01)	0.044
	Control (*n* = 293)	24.24 ± 3.82	24.81 ± 3.83	0.69 (0.59, 0.80)	< 0.001		
BMI z-score	Intervention (*n* = 390)	2.28 ± 0.79	2.27 ± 0.78	−0.02 (−0.05, 0.01)	0.314	−0.10 (−0.21, 0.00)	0.056
	Control (*n* = 293)	2.40 ± 0.82	2.40 ± 0.77	0.03 (0.04, 0.06)	0.026		
Fasting plasma glucose (mmol/L)	Intervention (*n* = 390)	4.77 ± 0.33	5.38 ± 0.42	0.61 (0.56, 0.64)	< 0.001	0.47 (0.42, 0.53)	< 0.001
	Control (*n* = 293)	4.72 ± 0.38	4.87 ± 0.41	0.15 (0.10, 0.20)	< 0.001		
Fasting insulin (μU/mL)	Intervention (*n* = 381)	13.32 ± 10.02	10.15 ± 7.43	** − 3.16 (− 4.07, − 2.25)**	**< 0.001**	** − 3.23 (− 4.46, − 1.99)**	**< 0.001**
	Control (*n* = 293)	15.31 ± 9.67	14.25 ± 10.81	− 1.05 (− 2.28, 0.18)	0.095		
^c^HOMA-IR	Intervention (*n* = 381)	2.86 ± 2.30	2.45 ± 1.85	** − 0.41 (− 0.63, − 0.19)**	**< 0.001**	** − 0.75 (− 1.17, − 0.33)**	**< 0.001**
	Control (*n* = 293)	3.23 ± 2.11	3.06 ± 2.39	− 0.17 (− 0.45, 0.11)	0.231		
Total cholesterol (mmol/L)	Intervention (*n* = 390)	4.16 ± 0.71	4.65 ± 0.79	0.49 (0.44, 0.55)	< 0.001	0.39 (0.31, 0.48)	< 0.001
	Control (*n* = 293)	4.24 ± 0.66	4.32 ± 0.71	0.07 (0.01, 0.14)	< 0.017		
Triglycerides (mmol/L)	Intervention (*n* = 390)	0.99 ± 0.48	1.06 ± 0.56	0.07 (0.02, 0.11)	0.004	0.12 (0.05, 0.18)	0.001
	Control (*n* = 293)	0.99 ± 0.45	0.94 ± 0.46	− 0.04 (− 0.09, 0.00)	0.072		
HDL-C (mmol/L)	Intervention (*n* = 390)	1.10 ± 0.23	1.45 ± 0.32	**0.34 (0.32, 0.36)**	**< 0.001**	**0.21 (0.18, 0.25)**	**< 0.001**
	Control (*n* = 293)	1.17 ± 0.24	1.28 ± 0.24	**0.11 (0.08, 0.14)**	**< 0.001**		
LDL-C (mmol/L)	Intervention (*n* = 390)	2.74 ± 0.80	3.50 ± 0.97	0.75 (0.68, 0.82)	< 0.001	0.23 (0.12, 0.34)	< 0.001
	Control (*n* = 293)	3.10 ± 0.77	3.54 ± 0.83	0.44 (0..35, 0.52)	< 0.001		
TG:HDL-C (mmol/L)	Intervention (*n* = 390)	0.95 ± 0.54	0.78 ± 0.48	** − 0.17 (− 0.21, − 0.12)**	**< 0.001**	− 0.02 (− 0.07, 0.04)	0.524
	Control (*n* = 293)	0.90 ± 0.48	0.77 ± 0.43	** − 0.12 (− 0.17, − 0.07)**	**< 0.001**		
Systolic BP (mmHg)	Intervention (*n* = 390)	98.69 ± 10.59	103.27 ± 10.04	4.57 (3.48, 5.67)	< 0.001	− 1.43 (− 2.86, 0.00)	0.050
	Control (*n* = 293)	102.97 ± 9.48	106.95 ± 9.90	3.97 (2.81, 5.14)	< 0.001		
Diastolic BP (mmHg)	Intervention (*n* = 390)	61.29 ± 9.64	61.3 ± 10.36	0.01 (− 1.25, 1.26)	0.990	** − 3.71 (− 5.24, − 2.18)**	**< 0.001**
	Control (*n* = 293)	66.42 ± 8.11	67.18 ± 8.67	0.75 (− 0.30, 1.81)	0.162		

Mean differences within and between groups are mean values, and negative changes indicate reduction from baseline to month 6

*Abbreviations*: *SD* standard deviation, *MD* mean difference, *BMI* body mass index, *HOMA-IR* homeostatic model assessment of insulin resistance, *HDL-C* high-density lipoprotein cholesterol, *LDL-C* low-density lipoprotein cholesterol, *TG:HDL-C* triglycerides to high-density lipoprotein cholesterol ratio, *BP* blood pressure

^a^Repeated measures analysis of variance (ANOVA) adjusted for gender, significant at *p* < 0.05

^b^Analysis of covariance (ANCOVA) adjusted for gender, significant at *p* < 0.05

^c^Adjusted for pubertal status

## Discussion

This study evaluated the cardiometabolic markers i.e. diabetes, insulin resistance, lipid profile, and blood pressure statuses of primary schoolchildren participating in a 6-month intervention study. The results showed the unexpected increase of fasting plasma glucose levels in the intervention group. However, the fasting plasma glucose levels are still within a normal range and of no clinical significance. The result might thus be attributed to the possibility that the children did not adequately fast before blood assessment as instructed. Although all precautions were taken, the fasting status of the children was confirmed verbally, and the instruction of overnight fasting might have not been respected. Similar results were observed in a previous school-based intervention, which showed higher prevalence of high fasting plasma glucose in both its intervention (+ 5.8%) and control (+ 7.0%) groups at the end of the assessment [32]. By contrast, a number of studies have shown an improvement in fasting plasma glucose after the intervention program [34, 35]. Despite the increment shown in fasting plasma glucose, fasting insulin and HOMA-IR showed a significant reduction in the intervention group, whereas a non-significant reduction was noted in the control group after six months of intervention. Other lifestyle intervention studies also reported reductions in insulin and/or HOMA-IR levels, which are consistent with our study [35–37].

The systematic review by Ho et al. [38] found fewer than half (5 out of 15) of the lifestyle intervention studies among overweight/obesity children resulting in a significant improvement of HDL-C or triglyceride levels. By contrast, our results showed a significant improvement in HDL-C levels as a result of an effective intervention, since the children enjoyed and followed the physical activities and nutrition education during the allocated sessions. This is supported by Cai et al. [39], who suggested that diet plus exercise interventions may exhibit a greater improvement in HDL-C than diet-only interventions.

Previous studies have indicated that one to three years of intervention consisting of physical activity and nutritional education gives positive effects on blood pressure in children [40, 41]. Unlike our study, children in the intervention and control groups exhibited a significant increment of systolic blood pressure, as seen in a 20-week exercise and diet guidance intervention on 19 overweight school-aged children [42]. In addition, previous studies by Guillaume et al. [43] and Klesges et al. [44] did not detect any reductions in systolic and diastolic blood pressure in physical activity intervention. These results have been attributed to the fact that blood pressure is consistent with aerobic abilities and maximum volume of oxygen (VO2 max), which are determined by the type and intensity of exercise rather than the amount of physical activity alone [45]. We suspect that better blood pressure outcomes may be obtained by using longer intervention periods similar to the observations by Hollar and Wang groups [40, 41].

Both intervention and control groups demonstrated increased levels of total cholesterol and LDL-C. In contrast to our study, a 10-week intervention developed by Slawta and colleagues [46] significantly decreased total cholesterol, LDL-C, and the ratio of total cholesterol and HDL-C. We postulate that this increment was due to school holidays and festive seasons which occurred during the study period, hence a longer duration of intervention may be needed to demonstrate a change in total cholesterol and LDL-C. Despite the increment of triglyceride levels after six months in the intervention group, the TG: HDL-C ratio showed an improvement, with higher reduction levels in the intervention group compared to the control group. This result is consistent with improved HOMA-IR. A recent study by Iwani et al. [14] showed that significantly higher numbers of overweight/obese children with a higher tertile of TG:HDL-C ratios had metabolic syndrome and insulin resistance. The improvement of TG:HDL ratio, hopefully would also signify an improvement in the number of children with metabolic syndrome.

A three-year intervention program, the Child and Adolescent Trial for Cardiovascular Health (CATCH) – the largest multi-component randomized controlled trial, despite having a school-based and family-based intervention, was unable to prove significant changes in blood pressure, anthropometry, and cholesterol measures, due to the inadequate statistical power and participation rates [47]. In the MyBFF@school study, we similarly did not observe improvement of certain cardiometabolic markers in the intervention group, despite having a good statistical power and participation rates. In fact, higher increments of cardiometabolic markers (fasting plasma glucose, total cholesterol, triglycerides, and LDL-C) were observed in the intervention group. This discrepancy may be attributed to the fact that the participants in the control group were voluntarily involved. Despite having no intervention (physical activity, nutrition, and psychology) being explicitly conducted in the control group, we postulate that they may have had more awareness of the purpose of this study and were motivated to change their lifestyle (physical activity and diet). Within the study itself, we had no control of this group and could not monitor their change of lifestyle. The increment of LDL-cholesterol levels might also indicate that children in the intervention group may have not practiced the nutritional education provided during the session at home, which again cannot be controlled, as it relates to the home environment. In addition, the nutritional intervention occurred in schools without direct involvement of the parents, limiting home-based monitoring. On top of that, children of this age do not always decide their own diet. Thus, parental support is important in achieving better health outcomes.

This study made several advancements. To our knowledge, MyBFF@school is the first large-scale school-based intervention study involving overweight and obese schoolchildren aged 9–11 years in Malaysia. The outcome of this study may help in narrowing the knowledge gap between childhood obesity and the reported associated increased risk of adult coronary heart disease and premature death [48–50]. The opportunity to obtain and analyze the blood samples of hundreds of children from this community-based research was also a strength of this study. Although some studies only include physical activity or nutritional education as their component of intervention, the MyBFF@school approach included elements of both with the addition of a psychology component.

The major limitation of the study is the short intervention period (six months). Hence, we were not able to evaluate the sustainability of the effectiveness of the program on clinical parameters and blood profile. As mentioned, the intervention (nutritional education, physical activity, and psychology sessions) occurred in schools without direct involvement of the parents, limiting home-based monitoring. In addition to that, children of this age do not always decide their own diet or know the recommended physical activity levels. Thus, parental support is important in achieving better health outcomes [51]. This is supported by a randomized controlled intervention involving 70 obese children aged 7–9 years that showed the effectiveness of group-based treatments involving parents and school nurses relative to individual counseling after one year [52]. Understanding children’s diet is a critical component when evaluating the success of a study focused on overweight/obese children. However, we did not measure calorie consumption before or after the intervention, and we acknowledge that this is one of the limitations of the study.

## Conclusion

The MyBFF@school intervention resulted in significant improvements of HDL-C, fasting insulin, and HOMA-IR in primary schoolchildren. However, increased in fasting blood glucose, total and LDL-cholesterol were seen. In view of the positive outcome, we recommend MyBFF@school program to improve the cardiometabolic markers of the overweight and obese primary schoolchildren. The addition of home-based intervention may further improve other cardiometabolic markers.

## Data Availability

All relevant data are within the paper.

## Abbreviations

ANCOVA: Analysis of covariance
ANOVA: Analysis of variance
BMI: Body mass index
HDL-C: High-density lipoprotein cholesterol
HOMA-IR: Homeostatic model assessment of insulin resistance
LDL-C: Low-density lipoprotein cholesterol
MyBFF@school: My Body is Fit and Fabulous at School
SD: Standard deviation
T2D: Type 2 diabetes
TG: HDL-C ratio: Triglycerides to high-density lipoprotein cholesterol ratio
WHO: World Health Organization
